# Supplementary material for: The Association between Utilization of Media Information and Current Health Anxiety Among the Fukushima Daiichi Nuclear Disaster Evacuees
Source: Int J Environ Res Public Health. 2020 Jun 1;17(11):3921. doi: 10.3390/ijerph17113921 (PMC7312024; doi:10.3390/ijerph17113921)
Supplement: Supplementary file 1 [file ijerph-17-03921-s001.pdf]

Supplementary Table S1. Characteristics of users of media relating to nuclear exposure (Forced evacuees)

Forced evacuees	Any local media (n=112) n (%)		Any national media (n=46) n (%)		Public broadcasting (NHK) (n=76) n (%)		Internet media (n=24) n (%)		Public relations from local governments (n=79) n (%)	
Age (as of August 2016)										
<40 years	10	(8.9)	4	(8.7)	6	(7.9)	7	(29.2)	1	(1.3)
40-64 years	55	(49.1)	20	(43.5)	29	(38.2)	16	(66.7)	41	(51.9)
≥65 years	47	(42.0)	22	(47.8)	41	(53.9)	1	(4.2)	37	(46.8)
Gender										
Male	43	(38.4)	18	(39.1)	29	(38.2)	13	(54.2)	35	(44.3)
Female	69	(61.6)	28	(60.9)	47	(61.8)	11	(45.8)	44	(55.7)
Education										
Junior/ Senior high school	82	(74.5)	32	(71.1)	55	(73.3)	10	(41.7)	60	(76.9)
Vocational college, University, Graduate school	28	(25.5)	13	(28.9)	20	(26.7)	14	(58.3)	18	(23.1)
Occupational category										
Employed or owner	43	(39.1)	9	(20.0)	27	(37.0)	14	(58.3)	25	(32.5)
Suspended from job	6	(5.5)	3	(6.7)	3	(4.1)	1	(4.2)	5	(6.5)
Unemployed	61	(55.5)	33	(73.3)	43	(58.9)	9	(37.5)	47	(61.0)

Supplementary Table S2. Characteristics of users of media relating to nuclear exposure (Voluntary evacuees)

Voluntary evacuees	Any local media (n=45) n (%)		Any national media (n=17) n (%)		Public broadcasting (NHK) (n=26) n (%)		Internet media (n=30) n (%)		Public relations from local governments (n=20) n (%)	
Age (as of August 2016)										
<40 years	8	(17.8)	5	(29.4)	1	(3.8)	13	(43.3)	4	(20.0)
40-64 years	24	(53.3)	7	(41.2)	14	(53.8)	16	(53.3)	6	(30.0)
≥65 years	13	(28.9)	5	(29.4)	11	(42.3)	1	(3.3)	10	(50.0)
Gender										
Male	19	(42.2)	5	(29.4)	11	(42.3)	11	(36.7)	10	(50.0)
Female	26	(57.8)	12	(70.6)	15	(57.7)	19	(63.3)	10	(50.0)
Education										
Junior/ Senior high school	25	(56.8)	7	(41.2)	15	(57.7)	10	(33.3)	12	(60.0)

Vocational college, University, Graduate school	19	(43.2)	10	(58.8)	11	(42.3)	20	(66.7)	8	(40.0)
Occupational category										
Employed or owner	30	(68.2)	8	(47.1)	19	(76.0)	25	(83.3)	13	(65.0)
Suspended from job	0	(0.0)	0	(0.0)	0	(0.0)	0	(0.0)	0	(0.0)
Unemployed	14	(31.8)	9	(52.9)	6	(24.0)	5	(16.7)	7	(35.0)

Supplementary Table S3. Prevalence of current health anxiety due to radiation exposure

	**Total** (n=223) n (%)		**Forced evacuees** (n=155) n (%)		**Voluntary evacuees** (n=68) n (%)		p value (χ^2)
Current health anxiety due to radiation exposure							
Not at all	28	(12.6)	21	(13.5)	7	(10.3)	
Only a little	47	(21.1)	32	(20.6)	15	(22.1)	
Somewhat	105	(47.1)	73	(47.1)	32	(47.1)	
Very	24	(10.8)	19	(12.3)	5	(7.4)	0.40
Extremely	19	(8.5)	10	(6.5)	9	(13.2)	(χ^2=4.05)
